# New Risk Methodology Based on Control Charts to Assess Occupational Risks in Manufacturing Processes

**DOI:** 10.3390/ma12223722

**Published:** 2019-11-11

**Authors:** Martin Folch-Calvo, Francisco Brocal, Miguel A. Sebastián

**Affiliations:** 1 Manufacturing and Construction Engineering Department, ETS de Ingenieros Industriales, Universidad Nacional de Educación a Distancia, Calle Juan del Rosal, 12, 28040 Madrid, Spain; msebastian@ind.uned.es; 2 Department of Physics, Systems Engineering and Signal Theory, Escuela Politécnica Superior, Universidad de Alicante, Campus de Sant Vicent del Raspeig s/n, 03690 Sant Vicent del Raspeig, Alicante, Spain; francisco.brocal@ua.es

**Keywords:** Bayesian inference, control chart, dynamic methodology, hidden Markov chain, occupational accident, risk assessment, risk control, risk management

## Abstract

The accident rate in the EU-28 region of the European Union showed a value of 2 fatal accidents per 100,000 people in 2019 that mainly affect construction (24%), manufacturing (19%) and logistics (19%). To manage situations that affect occupational risk at work, a review of existing tools is first carried out taking into account three prevention, simultaneity and immediacy characteristics. As a result, a new dynamic methodology called Statistical Risk Control (SRC) based on Bayesian inference, control charts and analysis of the hidden Markov chain is presented. The objective is to detect a situation outside the limits early enough to allow corrective actions to reduce the risk before an accident occurs. A case is developed in a medium-density fiberboard (MDF) manufacturing plant, in which five inference models based on Poisson, exponential and Weibull distributions and risk parameters following gamma and normal distributions have been tested. The results show that the methodology offers all three characteristics, together with a better understanding of the evolution of the operators in the plant and the safety barriers in the scenario under study.

## 1. Introduction

The accident rate in the European Union, [1] for the EU-28 region, was 2 fatal accidents per 100,000 people employed in 2015. The most affected industrial activities were: building (24%), manufacturing (19%), transport and storage (19%), agriculture—fishing (15%), retail (9%), public administration (9%), water supply–waste management (3%) and mining (2)%). In Spain, between 2014 and 2018, the most affected activities were practically the same [2].

**Figure 1:**
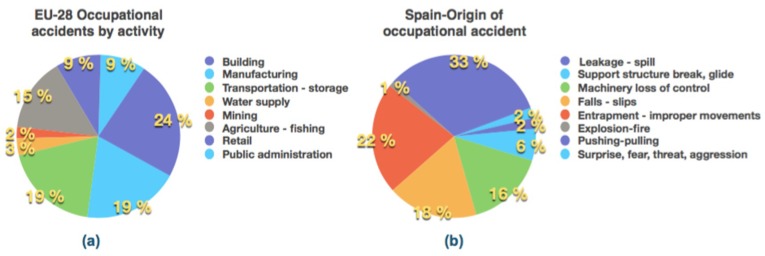
(**a**) Accident rate by activity in the year 2015 in the EU-28 countries of the European Union (adapted from [1]); (**b**) description of origin of occupational accidents in Spain for years 2014–2018 (adapted from [2]).

The main causes of accidents also in Spain for the same period 2014–2018 were: inadequate movements of the human body, in actions of pushing and pulling and by inappropriate body turns, all of them under physical effort (33%); entrapment and contact with sharp areas in machine elements (22%); falls and slips (18%); loss of total or partial control of a machine (16%); breakage and sliding of a work support (6%); leaks and spillages (2%); aggression (2%); and explosions–fire (1%) [2], Figure 1.

Of the total accidents generated by these causes, (99.1%) have had minor consequences, (0.8%) have caused severe damage, and (0.1%) have been very serious with a fatal outcome. Avoiding an accident at work regardless of its severity requires specific risk management, carried out throughout the life cycle; from design, engineering and construction, commissioning, operations, logistics and final dismantling; and in which at all times it is necessary to monitor qualitatively and quantitatively the moment in which an accident risk arises.

There are different points of view about the concept of risk; for example, in general a risk arises from the existence of uncertainty [3], in a more specific way a risk arises from the existence of uncertainty in the objectives [4,5], or in more practical terms, risk can be defined as the measure of lack of security [6]. The idea of security also has different meanings that can be defined simply as the lack of accidents, or from a labor point of view, how people can provide the required performance in expected and unexpected conditions [7], or quantifying safety as a numerical condition where the number of adverse outcomes is acceptably small [8] or from an analytical point of view that defines it as the study of why things go wrong [9].

Despite the definitions, the most important issue is how to manage risk in various scenarios and specifically those related to occupational safety at work. In this sense, an initial framework issued by the European Union is Directive 89/391/EEC [10] aimed at employers to establish basic principles of prevention with risk assessment and its avoidance being a preliminary and basic proposal to carry out an assessment of occupational risks.

The ISO 31000:2018 establishes the principles of risk management and the ISO/IEC 31010:2019 establishes the risk management-assessment associated techniques. It is based on the Deming cycle [11], consisting of a sequence of steps: “plan, do, check, act”.

**Figure 2:**
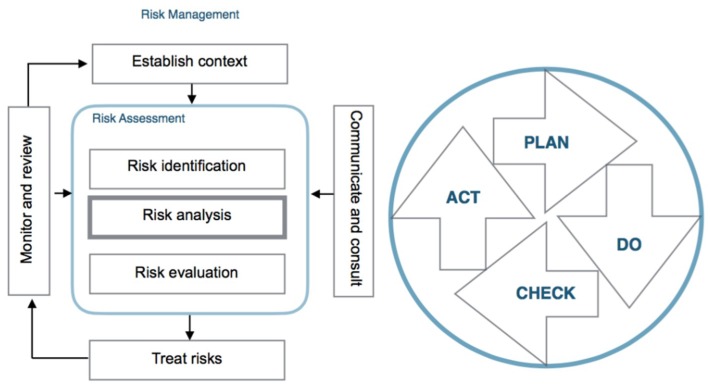
Risk-management process and Deming-cycle equivalence (adapted from [5]).

Quantitative risk assessment (QRA) is a formal and systematic risk-analysis approach to quantifying the risks associated with the industrial and human processes. The risk assessment is the general procedure that covers the risk identification process which can be performed based on historical data, through a panel of experts or using inductive cause-effect techniques; its analysis applying: qualitative methods, indicating the levels of importance of the risk and its consequences; semi-quantitative methods, indicating numerical risk rating scales and their consequences; and quantitative methods, defining the probabilities of risk-generation and its consequences [12,13,14], and their evaluation that implies determining the importance and prioritizing from the point of view of risk consequence or benefit–cost [15], Figure 2.

With the objective of managing occupational hazards, the ISO 45001 guideline [16] aims to provide guidelines that allow the implementation of a system of occupational health and safety (OH&S). In Spain, the main guidelines on occupational hazards come from the National Institute of Occupational Safety and Health, and are issued with the objective of providing guidelines that allow analyzing and studying health and safety conditions in the workplace, as well as their improvement [17]. In the context of manufacturing processes involving chemical agents, there are two European directives on the assessment of chemical agents at work [18] and for carcinogens and mutagens at work [19].

In this context, is especially important to prevent both occupational accidents and major accidents which can be interrelated [20], by means of adequate management and assessment methodologies. In the scientific literature, methodologies oriented to the management and assessment of occupational health and safety risks are collected with the addition of specific tools; such as a risk assessment based on fuzzy logic with application in the mining industry [21] and manufacturing plants [22]; or the two cases of risk assessment in process industries and to detect and evaluate emerging risks in industrial processes [23,24]; or the application of a multi-objective evolutionary algorithm to take into account the tasks, activities, associated risks and safety of workers with case studies associated with scaffolding falls [25,26]; the use of Bayesian networks applied to work situations on the high seas where slips, trips and falls must be avoided [27]; several cases of risk assessment applied in the mining industry [28]; in situations of construction of scaffolding falling objects and contact with moving machines and vehicles [29]; risk assessment in an aluminum process industry for workers using press extruders, forklifts, cranes, and production [30]; and the use of block diagrams to identify and evaluate fall hazards from an escalator [31]. It is applied in the construction of a natural gas pipeline using a tool based on Pythagorean fuzzy logic [32], the risk assessment produced by falls and falling objects, crane handling, and interaction with the moving parts of the machines [33,34,35,36], and risk assessment in occupational accidents related to the construction, operation and maintenance of wind farms on land [37].

However, there is no method that allows us to obtain an overview of the state of occupational risk in a manufacturing plant in the simplest way possible that is at the same time formal, which warns about the existence of a risk to avoid in advance. For this, three characteristics are considered necessary:

1. Prevention (P): be the process of avoiding or mitigating risks by reducing their probability of occurrence and their impacts on human and social; geographical and landscape; economic and infrastructure; environmental and ecosystem preservation; accident and safety (human, assets, production); perception and expectations.
2. Simultaneity (S): is the ability to update the evolution of risk according to the operation in real time.
3. Immediacy (I): is the ability to inform or infer the existence of a risk with sufficient anticipation to make the necessary corrections before the accident occurs.

To achieve this objective, it is necessary to first review what are the current tools related to risk management and evaluation and if, verifying which meet the three characteristics. Based on the most appropriate methodology, the objective of this paper is to present a new tool called Statistical Risk Control (SRC) for to manage and assess the situations of risk, focused in occupational accidents. With this object, this work is organized as follows: in Section 2 we review the state of the existing tools; in Section 3 we examine the development of the SRC methodology applied for occupational accidents; in Section 4 we present a case study and the results; in Section 5 we discuss the results; and in Section 6 we draw conclusions.

## 2. Existing and Related Tools for Occupational Risk Management

### 2.1. Regulations and Traditional, Modern Models

The analysis covers three main groups:

1. The first group corresponds to the standards and directives whose characteristics and degree of compliance with the three characteristics (P), (S), (I) are summarized in Table 1.
2. The second group covers methodologies and models differentiating the traditional and the modern approaches [38]. The traditional approach includes the sequential and the epidemiological models, summarized in Table 2. The modern approach has five models: the systematic; cloud based; the fuzzy based, formal based and safety barrier based; summarized in Table 3.
3. The third group, which is encompassed in the modern methodologies, is specific for dynamic models and it is discussed in the next subsection.

### 2.2. The Dynamic Risk Models

This group covers a dynamic risk-analysis concept using sequential models like fault-tree analysis (FTA), event-tree analysis (ETA) and the BOW-TIE graph approaches and performing a Bayesian inference analysis to update the failure probabilities from the information collected of the named accident precursors or precursor data. This group has five models; the dynamic risk assessment (DRA) being the representative; the dynamic procedure for atypical scenarios identification (DyPASI), the dynamic risk analysis, the risk barometer methodology and the dynamic operational risk assessment, [86,87,88].

DRA is an extension of the risk assessment (RA). The process needs to establish a prior function for the statistical parameter that models the risk probability. The precursors, events or causes that can lead to an accident are observed and formalized through the application of Bayesian inference to obtain the posterior function of the parameter that models the risk probability, [89,90,91,92,93,94].

**Figure 3:**
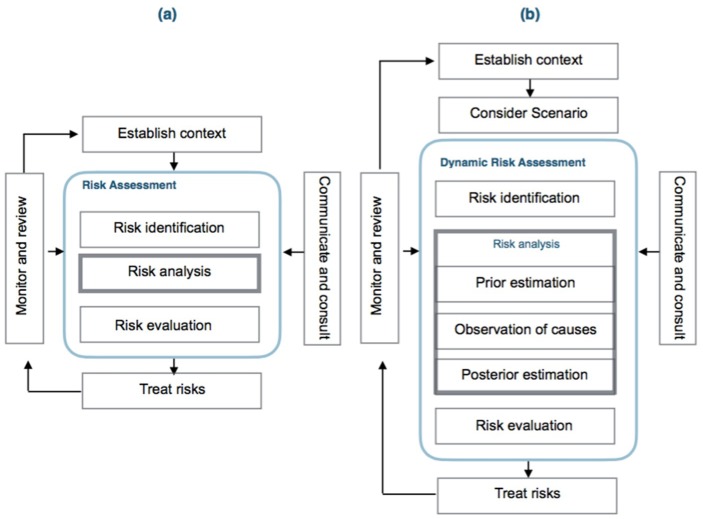
(**a**) Risk assessment; (**b**) dynamic risk assessment (adapted from [5]).

Figure 3 presents the main equivalences between RA and DRA models. Highlights are:

- Risk identification is similar as presented in the ISO/IEC 31010:2019. The identification of potential risks are performed by the application of HAZOP/HAZID and FMEA, FMECA techniques [95].
- Scenario consideration is similar to the answer to the “What if?” question. Scenarios reflecting the “best case”, “worst case” and “expected case” may be used for quantifying the probability of potential consequences and obtain a sensitivity analysis.
- After identification of causes of risk, their paths and sequences through the safety barriers are defined using the ETA or FTA methods under a bow-tie graph and ending at the final states. Reliability data bases can be applied for human, equipment or barriers failure, or using expert judgment [96,97].
- Observation of precursor data, events and situations from the workplace or the process under analysis.
- Posterior estimation is performed using Bayesian inference through the expression:

*f*(*p*/*Data*)∝*g*(*Data*/*p*)·*f*(*p*) (1)

where *p* is the statistical parameter, *f*(*p*) is the prior statistical distribution for the parameter *p*; *g*(*Data*/*p*) is corresponding to the observed precursor data and *g*(*p*/*Data*) is the posterior statistical distribution.

This strategy has been applied to a number of case studies in petrochemical industry including the case for a storage tank containing hazardous chemicals, a refinery, and oil-spill accidents; or additionally performing the inference using Bayesian Networks applied in offshore oil and gas accidents [98,99,100,101,102,103].

The characteristics for the four remaining models can be seen in Table 4.

## 3. Statistical Risk Control (SRC) Methodology

### 3.1. General Application

**Figure 4:**
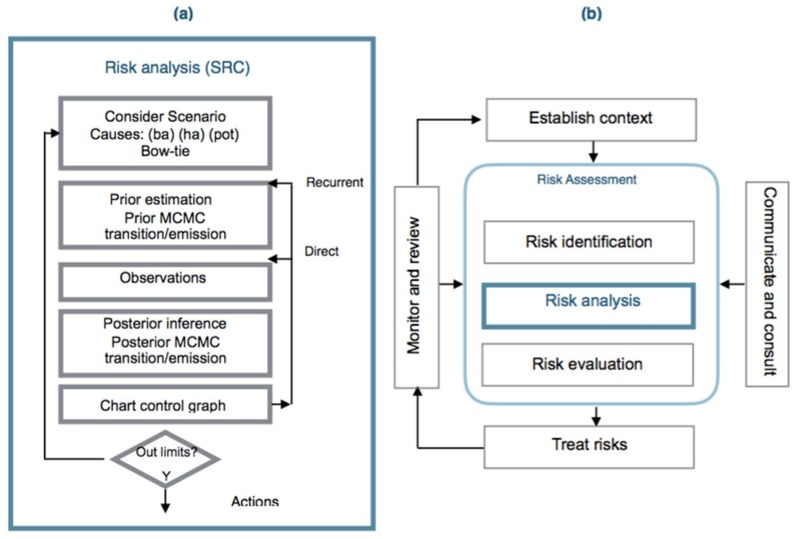
(**a**) Statistical Risk Control (SRC) methodology; (**b**) position in the risk-assessment scheme (adapted from [5]).

There is a need to use control charts and a dynamic risk assessment model for analyze risk in general industrial situations. There is no literature in the use of the control charts for risk management but nevertheless they are applied in the earned value method in project management [109], in environmental assessment [110] or for cost control and project duration [111]. The methodology is compatible with the ISO guidelines using the Bayesian inference and Hidden Monte Carlo–Markov methods under a new concept of Statistical Risk Control (SRC) [112] (Figure 4).

**Figure 5:**
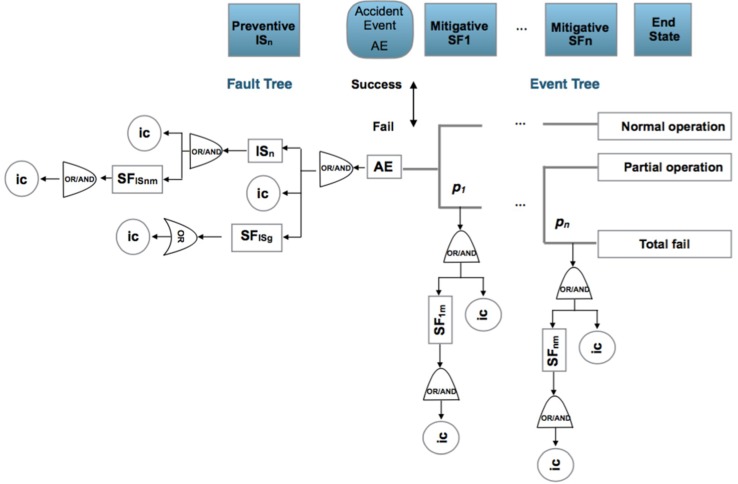
Safety barriers: IS_n_: preventive barrier (n); SF_ISnm_: preventive barrier (n) sub-function (m); SF_ISg_ general sub-function safety barrier; SFn: mitigative barrier (n); SF_nm_: mitigative barrier (n) sub-function (m); ic: initiation causes (ba, ha, pot); p_,s,f_: probability of success or fail in the mitigative or reactive safety barrier.

When the risk has been identified in a considered scenario, the following seven steps are applied:

1. A bow-tie analysis is performed to provide a visual representation of the causes of initiation (ic) classified as basic, human and potential that affect preventive and mitigative safety barriers and the consequences or final states when an accident occurs, Figure 5.
2. The identification and definition of the initiation causes (ic) which may be: basic events (ba) such as failures in control systems, equipment or processes; human risk factors (ha) which are human errors and the potential causes (pot), which will be defined in the following subsection. The process is iterative between step 1 and step 2 until the causes and consequences have been clearly established.
3. From the previous steps 1 and 2, the statistical parameter *p* that expresses the risk probability is also identified and the prior statistical distribution that reflects it can be established. Also the prior transition and emission matrices governing changes in the mitigative safety barriers can be defined.
4. The observation of the initiation causes (ic) and end states are put into effect according to a time interval.
5. From the estimated prior *f*(*p*) and the observed initiation causes (ic), such as *g*(*data*/*p*) and applying Equation (1), the posterior function for the statistical parameter (*p*) can be obtained and if there is not an analytical expression for it then the Metropolis–Hastings sampling method can be applied to obtain the posterior distribution and its associated parameters [113,114]. Also corresponding to the hidden Markov chain, the prior transition and emission matrices are defined for the mitigative safety barriers and the posterior transition and emission matrices are obtained using the Baum–Welch algorithm, [115,116].
6. Control chart presentation [117,118] to graph the evolution of the statistical parameter *p*, in a time interval.

Chart determination has two modes:

- Direct: uses the observed data up to the analyzed interval time, but with two possibilities: the mean established in the prior function that defines the statistical parameter (*p*) is constant in every interval, and the standard deviation is determined using the observed data collected up to the analyzed interval, (Direct–Mean Prior) or by modifying the mean and the standard deviation also using the observed data collected up to the analyzed interval (Direct–Mean Posterior).
- Recurrent: uses the observed data in every interval time, also with two possibilities: maintaining the mean posterior constant, and the standard deviation obtained in every interval is the new prior in the following interval (Recurrent–Mean Prior), or the mean and the standard deviation obtained in every interval are the prior values in the following interval (Recurrent–Mean Posterior).

**Figure 6:**
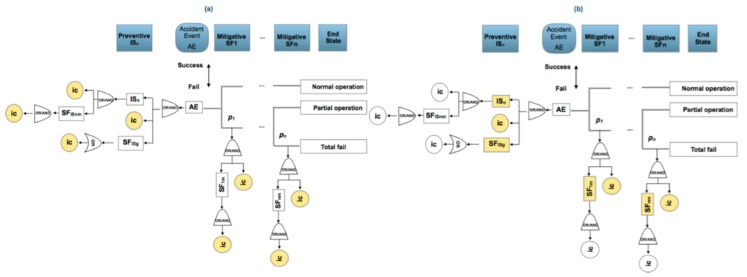
Analysis modes. (**a**) collecting total of (ic’s) of preventive and mitigative barriers and their sub-functions (highlighted yellow); (**b**) collecting at the first level of (ic´s) and barriers sub-functions (highlighted yellow).

**Figure 7:**
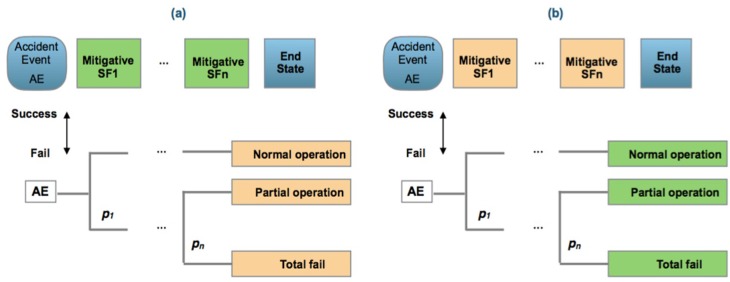
Analysis modes of the hidden Markov chain. (**a**) Defining transition probabilities for the mitigative barriers (highlighted green) and emission probabilities for the observed end sates in function of the mitigative barriers (highlighted brown); (**b**) defining transition probabilities for the end states (highlighted green) and emission probabilities for the observed mitigative barriers states (highlighted brown).

Considering also the observed initiation causes (ic), and visualizing the bow-tie, there are two possibilities of analysis to include in the control chart:

- For the complete bow-tie scheme, Figure 6.
  a.1. Collecting the total of the initiation causes (ic) affecting all the preventive and mitigative safety barriers and their barrier sub-functions.
  a.2. Collecting only the first level for initiation causes (ic) and fails in first level of barrier sub-functions.
- Observing the fault tree (FT) and event tree (ET), and analyzing the response active (yes) or (no) for the preventive and mitigative safety barriers.

7. Analysis applying a hidden Monte Carlo Markov Chain for the mitigative safety barriers, also with two possibilities, Figure 7.
  - Analyzing the behavior of the mitigative safety barriers based on the end states. In this case a transition matrix is defined for the mitigative barriers and an emission matrix for the observed end states in function of the barriers’ transition.
  - Analyzing the behavior of the end states based on the action of the mitigative barriers. In this case a transition matrix is defined for the end sates and an emission matrix for the observed mitigative safety barriers in function of the end states.

The Baum–Welch algorithm is applied to obtain, from the observations, the posteriors transition and emission matrices with a methodology that can also be direct or recurrent.

### 3.2. Potential Causes (Pot)

**Figure 8:**
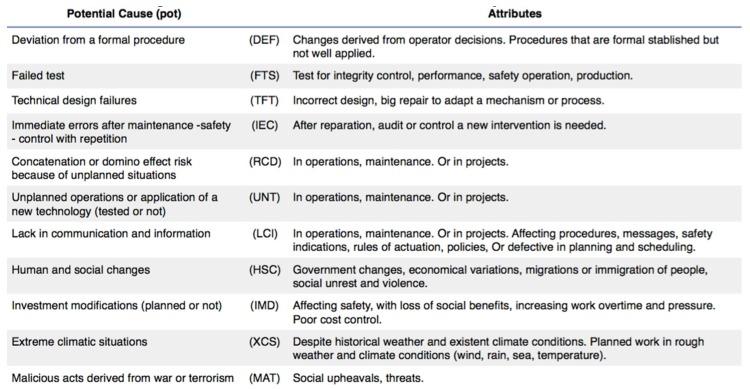
Potential causes (pot) and attributes defined in the Statistical Risk Control (SRC) methodology.

Some authors consider [119] that when unexpected deviations arise due to causes that are difficult to predict because of their randomness, this can be classified as a special risk [120,121]. From the point of view of the methodology (SRC), these causes that can lead to unexpected risk situations are called potential causes, which are summarized in Figure 8.

### 3.3. General Application for Occupational Accidents

**Figure 9:**
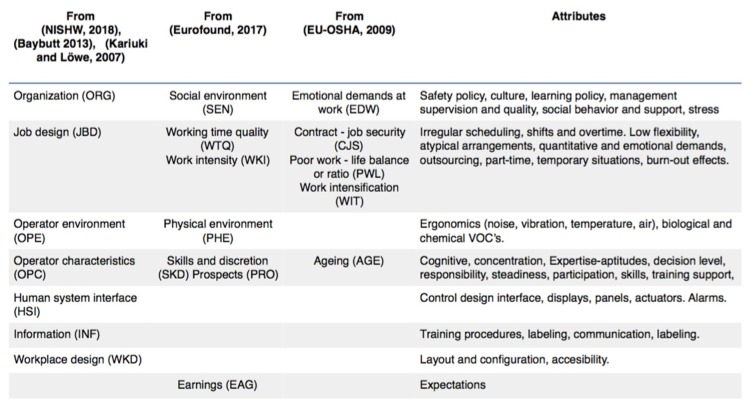
Factors and attributes affecting human behavior at work.

In accordance with the SRC methodology, a bow-tie is defined in each particular scenario. In the case of occupational accidents before being able to work with different risk scenarios, it is necessary to analyze what human behavior is at work and what are the factors that affect it. Three views and organizations that cover the most critical have been considered. The first group compiled is general, [17,122,123]. The second group includes the factors considered critical in the 6th European Survey of Working Conditions [124], and the third group includes the emerging psychosocial risks related to occupational safety and health [125]. The aggregation and coincidence of the three groups is presented in Figure 9.

**Figure 10:**
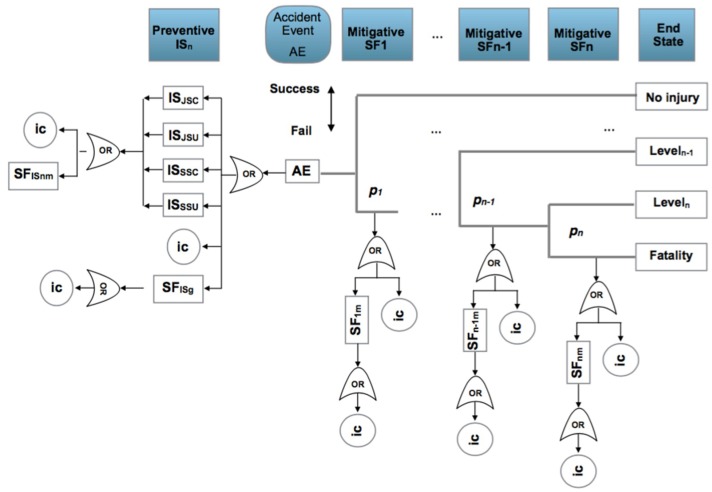
General bow-tie for occupational accidents. Safety barriers: IS_n_: preventive barrier (n) based on job self control (JSC); job supervision (JSU); safety self control (SSC) and safety supervision (SSU). SF_ISg_ general sub-function working parallel to the human actions; SFn: mitigative barrier (n); SF_nm_: mitigative or reactive barrier (n) sub-function (m); ic: initiation causes (ba, ha, pot); p_,s,f_: probability of success or fail in the mitigative or reactive safety barrier.

These critical factors must be taken into account as possible causes of initiation (ic) of an occupational accident and must be present in all scenarios. However, it is considered that there are four situations that occur as a consequence and symptom of the critical factors. These are: failure in the self-control of work (JSC); failure to supervise work (JSU); failure in security self-control (SSC) and failure in security supervision (SSU). In addition, these four situations that are easily observable arise in an automated process and manufacturing environments, where workers additionally perform control and supervision tasks. These four situations, pro their control and supervision function, are associated with safety barriers. The general bow-tie for occupational accidents (Figure 10), is equivalent to the general one presented above, with the differences in preventive barriers (IS_n_) integrated by the four safety barriers (IS_JSC_, IS_JSU_; IS_SSC_; IS_SSU_). In this scheme, the (SF_ISnm_) are sub-functions of the four safety barriers, which can be formed by a procedure, an automatism, an alarm indication, an actuator of a control system or the organizational culture itself. The general safety barrier (SF_ISg_) works in parallel with the activity of the operators and may consist of automatic control systems, protections, alarms, actuators or automated operations management that guides the operator at each step of the process and does not permit the next step to be formalized if a number of conditions are not met. Sub-function barrier (SF_nm_) covers the various functional components that integrate the mitigating safety barriers. The final states represented are bounded at one end by the absence of personal injuries, if the first mitigating safety barrier acts correctly and the other by a fatality if the failure of all mitigating safety barriers occurs. The graph must be taken as a framework and must adapt according to the processes, the occupational works and the scenarios that are being analyzed.

## 4. Case Study in a Medium-Density Fiberboard (MDF) Manufacturing Process Plant

### 4.1. Process

**Figure 11:**
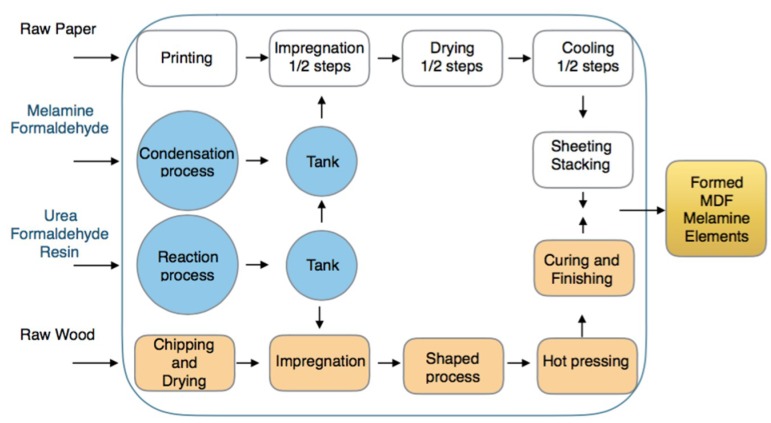
General production scheme of a medium-density fiberboard (MDF) urea-melamine plant.

The general process scheme is depicted in Figure 11. Its goal is to produce urea-melamine medium density fiber (MDF) board elements using as basic raw materials paper, wood, melamine, urea, a resin (such as a polyamide or vinyl chloroacetate) and formaldehyde. The paper is subjected to a surface printing treatment continuing with the impregnation phase performed with melamine-formaldehyde. Drying and cooling processes are executed next in a single step if only the melamine-formaldehyde polymer is added or with one additional step if the urea-formaldehyde polymer is added, and with the same impregnation, drying and subsequent cooling steps. The process continues with the cutting and winding of the paper and its stacking. At the same time, the wood is splintered by subsequently drying the material at 180 °C to reduce moisture. The dry material (8% moisture) is impregnated with the urea-formaldehyde solution and the resin. It follows a stage of forming and pressing at 200 °C. The board thus obtained is subjected to a curing process, and is completed in a union-pressing stage of the board formed with the sheet of paper.

In plant there are 42 workers distributed in two shifts. The plant is highly automated with robots for handling, feeding, palletizing and control systems in every step. The finishing area is made up of panel sectioning machines composed of vertical bar and pressure sawing machines, as well as circular saws of one or several discs, in addition to a final sanding and calibration zone. Also in the work areas and in order to maintain the correct level of particles and volatile organic compounds (VOC) emissions, there is a centralized air aspiration system with subsequent filtration an purification processes prior to its emission to the environment. Quality and safety policies are established. Workers wear personal protective equipment and there are periodic safety checks at the process plant.

### 4.2. Results

**Figure 12:**
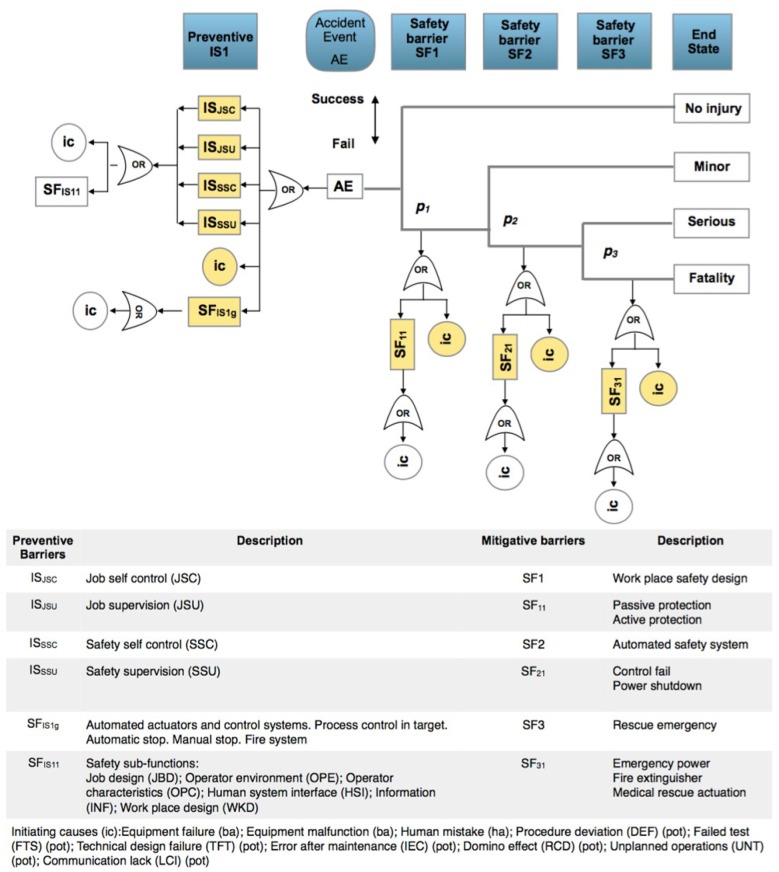
Bow-tie for occupational accident first level analysis in the MDF process plant.

The analysis covers the general plant and the bow-tie is presented in Figure 12 with four final states: no injuries, minor, serious, and fatal. If an accident event (AE) is generated, the mitigating safety barriers (SF_1_ SF_2_ SF_3_) are activated. The final states represented are bounded at one end by the absence of personal injuries, if the first mitigating safety barrier (SF1) acts correctly, in case of failure the second barrier (SF2) acts ending with a minor injury if it works correctly; in case of failure the third barrier (SF3) acts, leading to a serious problem in case of correct operation, or to a fatality in case of failure. The sub-functions (SF_11_ SF_21_ SF_31_) correspond to automatisms, procedures, alarms and active or passive protections belonging to the main function of each of the mitigating safety barriers.

**Figure 13:**
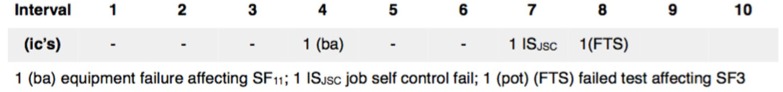
Initiation causes (ic´s) and barrier fails collected at 10 intervals.

The analysis is carried out at the first level, highlighted in yellow on the graph. The observations are made in the worst case taking the plant in general, which means that the observations are collected on the one hand for the preventive barriers, the general safety barrier (SF_ISg_) and the initiation causes (ic) that collect all workers in one shift; and on the other hand, from each of the mitigative barriers. SF1–SF_11_ and associated (ic) is the group in which the general passive and active protections are located in each workplace of the plant; the SF2-SF_21_ and associated (ic) is the group in which the general safety controls, fire extinction, alarms and the automated or manual shutdown of every workplace are located, and the SF3-SF_31_ and associated (ic) is the group for the general emergency power, general fire extinction systems and internal rescue actuations. The observations are made at 10 time intervals per day, covering all shifts. Figure 13 shows three observed causes in intervals 4, 7 and 8.

Collecting observations is expected that can follow a Poisson, an Exponential or a Weibull distributions, being the *g*(*data*/*p*) in Equation (1). And the statistical parameter *p* is corresponding to the rate λ or frequency of events and the prior *f*(*p*) can be defined as a gamma or a normal distribution.

#### 4.2.1. Poisson–Gamma Model

With a Poisson–gamma model the expression for *f*(*p*/*data*)∝*g*(*data*/*p*)*·f*(*p*) with parameter *p* = λ is;

*f*(*λ*/*data*)∝*g*(*data*/*λ*)*f*(*λ*)∝*λ*^(*α*+s)−1^e^−^*^λ^*^(β+N*t*)^= *gam*(α +s,β +N*t*) (2)

where;

*s* = *∑data* = *∑y_i_* (3)

Being *Nt* number of interval and *s* the sum of initiation causes (ic’s) and safety barriers incidences as data *y_i_* in the corresponding time interval *i*. The values α and β are the parameters of the gamma distribution.

**Figure 14:**
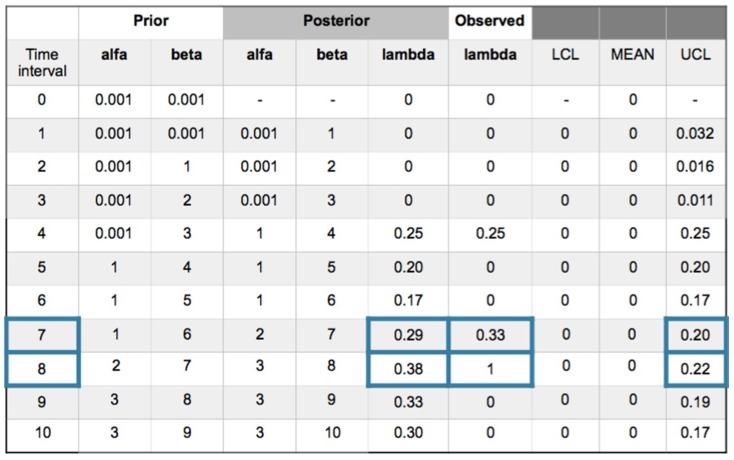
Poisson–gamma model. Recurrent with mean prior method. Evolution of collected (ic’s) and safety barrier fails in 10 intervals.

A recurrent method with mean prior and equal to a desired value as a target, is applied. In this case the target is for have zero accidents, then the parameters of the gamma prior are α = β = 0.001. Working with +/−1σ_post_ the posterior values are (Figure 14):

The following comments can be made for every interval.

Interval 1. With zero incidences, is, *y_i_* = [0] and posterior density for λ; *gam*(*α* + *s*, *β* + *Nt*) = *gam*(0.001, 1) with λ_post_ = 0 and σ_post_ = 0.031.

Interval 2. Also with zero incidences, is, *y_i_* = [0] and posterior density for λ; *gam*(*α* + *s*, *β* + *Nt*) = *gam*(0.001, 2) with λ_post_ = 0 and σ_post_ = 0.016.

Interval 3. With zero incidences, is, *y_i_* = [0] and posterior density for λ; *gam*(*α* + *s*, *β* + *Nt*) = *gam*(0.001, 3) with λ_post_ = 0 and σ_post_ = 0.011.

Interval 4. With one incidence in one sensor affecting the SF_11_ safety barrier sub-function, is in this case, *y_i_* = [1] and posterior density for λ; *gam*(*α* + *s*, *β* + *Nt*) = *gam*(1, 4) with λ_post_ = 0.25 and σ_post_ = 0.25. Due to the no memory characteristic of the exponential, Poisson and Weibull distributions, the observed parameter value for 1 incidence in 4 time intervals is 0.25 that is coincident with the posterior. The values are in the upper control limit (UCL).

Interval 5. With zero incidences, is, *y_i_* = [0] and posterior density for λ; *gam*(*α* + *s*, *β* + *Nt*) = *gam*(1, 5) with λ_post_ = 0.20 and σ_post_ = 0.20. As a characteristic of the Bayesian inference, the posterior distribution responds softening the reduction of parameter λ from 0.25 to 0.20.

Interval 6. With zero incidences, is, *y_i_* = [0] posterior density for λ; *gam*(*α* + *s*, *β* + *Nt* = *gam*(1, 6) with λ_post_ = 0.17 and σ_post_ = 0.17. The Bayesian inference also responds by softening the reduction of parameter λ from 0.20 to 0.17.

Interval 7. With one incidence affecting a job self control fail (JSC) in the hot pressing process, with *y_i_* = [1] and posterior density for λ; *gam*(*α* + *s*, *β* + *Nt*) = *gam*(2, 7) with λ_post_ = 0.29 and σ_post_ = 0.20. The observed parameter value for 1 incidence in 7 − 4 = 3 time intervals is 0.33 that is practically coincident with the posterior. An out of limits is also displayed.

Interval 8. With one incidence affecting a failed test in the SF3 safety barrier, with, *y_i_* = [1] and posterior densityfor λ; *gam*(*α* + *s*, *β* + *Nt* = *gam*(3, 8) with λ_post_ = 0.38 and σ_post_ = 0.22. The observed parameter value for 1 incidence in 8 − 7 = 1 time intervals is 1. And also shows and out of limits.

Interval 9. With zero incidences, is, *y_i_* = [0] and posterior density for λ; *gam*(*α* + *s*, *β* + *Nt* = *gam*(3, 9) with λ_post_ = 0.33 and σ_post_ = 0.19.

Interval 10. With zero incidences, is, *y_i_* = [0] and posterior density for λ; *gam*(*α* + *s*, *β* + *Nt* = *gam*(3, 10) with λ_post_ = 0.30 and σ_post_ = 0.17.

**Figure 15:**
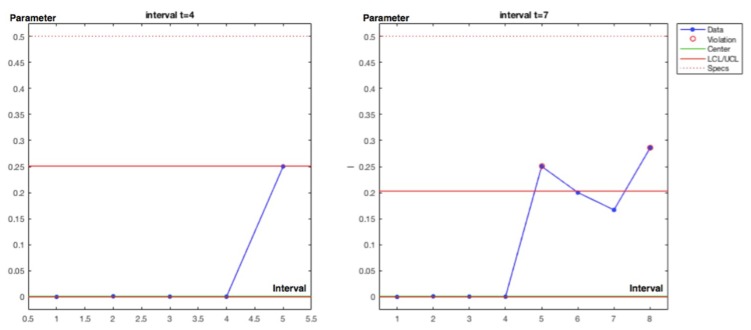
Poisson–gamma model. Recurrent with mean prior method. Charts based on posterior lambda evolution for intervals 4 and 7.

**Figure 16:**
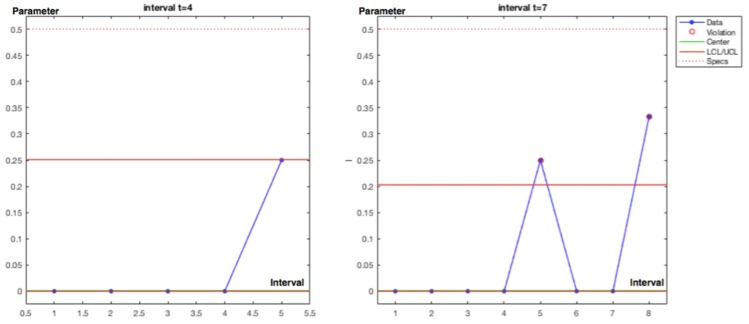
Poisson–gamma model. Recurrent with mean prior method. Charts based on observed lambda evolution for intervals 4 and 7.

Charts for intervals 4 and 7 for posterior and observed values are presented in Figure 15 and Figure 16.

#### 4.2.2. Exponential–Gamma Model

With an exponential–gamma model the posterior expression *f*(*p*/*data*)∝*g*(*data*/*p*)*·f*(*p*) with parameter *p* = λ is:

*f*(*λ*/*data*))∝*g*(*data*/*λ*)·*f*(*λ*)∝λ^α^ e^−λ(β+t)^ (4)

being α and β the parameters of the gamma distribution and *t* the observed time between causes.

**Figure 17:**
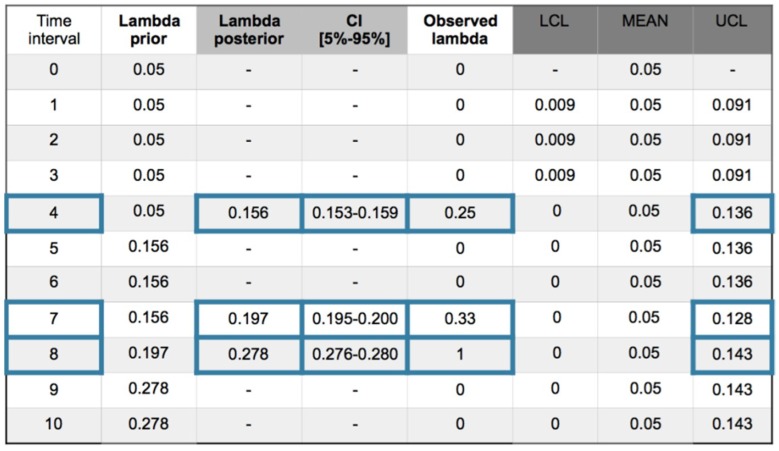
Exponential–gamma model. Recurrent with mean prior method. Evolution of collected (ic’s) and safety barrier fails in 10 intervals. Confidence interval (CI) [5%–95%]. Out of limits framed blue.

**Figure 18:**
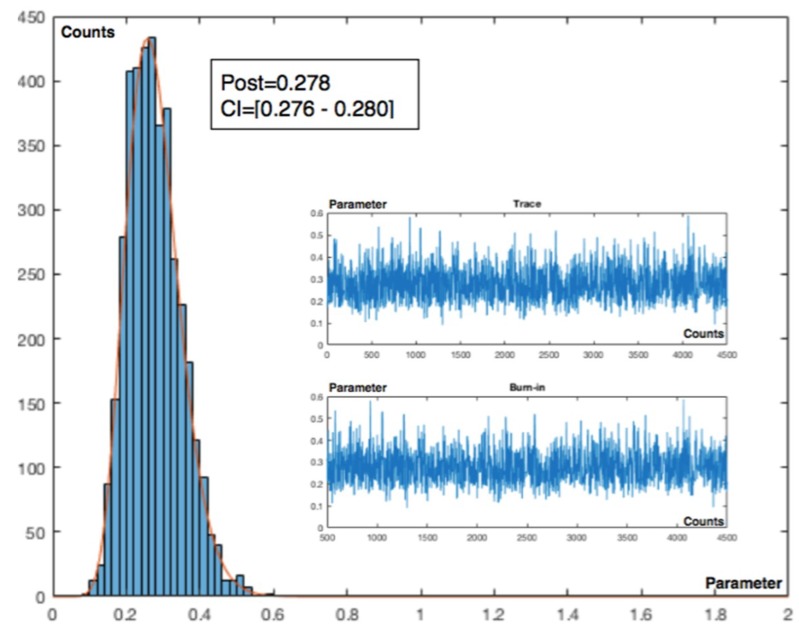
Metropolis–Hastings. Interval 8. Sampling n = 4500, burn = 500; 10 cycles. Acceptance rate (AR) = 58%.

The analysis also will be effectuated using a recurrent method with mean prior and equal to a desired value as a target, being in this example changed to a less restrictive value of 1 accident in 20 time intervals and equal to 0.05, with gamma prior parameters α = 0.5 and β = 10. Working with +/−1σ_post_ and using the Metropolis–Hastings (MH) sampler, Figure 17 shows the collected values and the (MH) sampling with acceptance rate (AR) 58% at interval 8 in Figure 18.

#### 4.2.3. Weibull–Gamma Model

With a Weibull–gamma model the human fatigue is considered in the analysis and the posterior expression for *f*(*p*/*data*)∝*g*(*data*/*p*)*·f*(*p*) with parameter *p* = λ is;

*f*(*λ*/*data*))∝*g*(*data*/*λ*)*·f*(*λ*) ∝ (*λ*)*^α^*^−1^·*exp* (−*λβ*)·(*λ*)*^c^·exp* (−(*λt*)*^c^*) (5)

being α and β the parameters of the gamma distribution, *t* the observed time between causes and c is the fatigue parameter.

**Figure 19:**
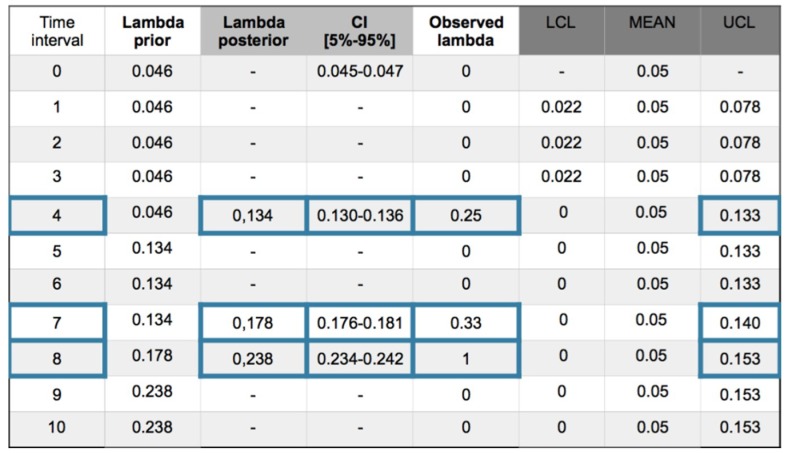
Weibull–gamma model. Recurrent with mean prior method. Evolution of collected (ic’s) and safety barrier fails in 10 intervals. Confidence interval (CI) [5%–95%]. Out of limits framed blue.

**Figure 20:**
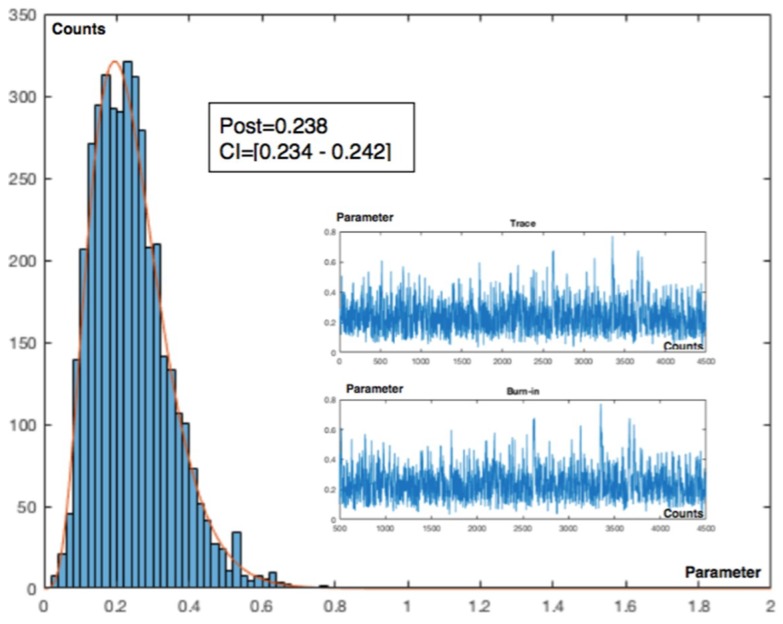
Metropolis–Hastings. Interval 8. Sampling n = 4500, burn = 500; 10 cycles. AR = 63%.

When c = 1 the failure rate function is constant being equivalent to an exponential–uniform model. If c > 1 the failure rate function is increasing. If 0 < c < 1 the failure rate function is decreasing. A conservative c = 1.5 value can be adopted. With a value of 1 accident in 20 time intervals equal to 0.05 and working with +/−1σ_post_ the Metropolis–Hastings sampler is applied. The Figure 19 shows the collected values and the (MH) sampling with acceptance rate (AR) 63% at interval 8 in Figure 20.

The out of limits are presented in the same time intervals as the previous model. It should be noted that the parameter *p* = *λ* is modeled as a gamma distribution. The posterior distribution is also a Gamma distribution independently of the observations as Poisson, exponential or Weibull functions. Another possibility is to apply a normal distribution for the approximation to the parameter *p* characteristic.

#### 4.2.4. Exponential–Normal Model

With the exponential–normal model the posterior expression *f*(*p*/*data*)∝*g*(*data*/*p*)*·f*(*p*) with parameter *p* = λ is;

*f*(*λ*/*data*))∝*g*(*data*/*λ*)*·f*(*λ*)∝λ*exp* (−λ*t*)∙*1*/σ *exp* (−(*x* − λ)^2^/*2σ*^2^ (6)

being *t* the observed time between causes and *x* the evolution of the parameter value.

**Figure 21:**
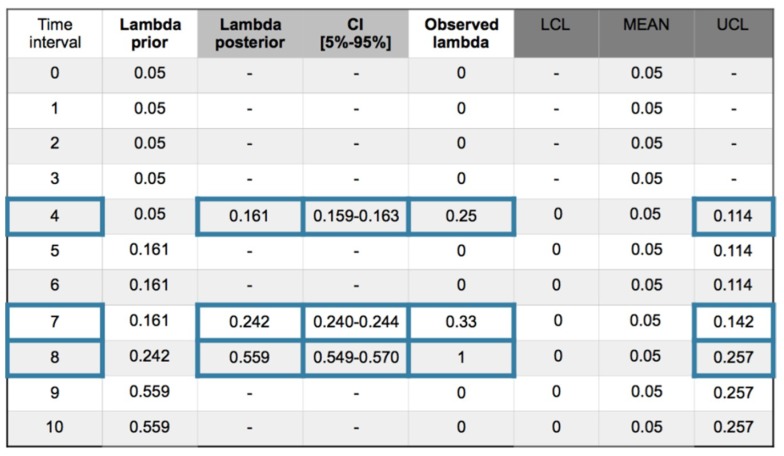
Exponential–normal model. Recurrent with mean prior method. Evolution of collected (ic’s) and safety barrier fails in 10 intervals. Confidence interval (CI) [5%–95%]. Out of limits framed blue.

**Figure 22:**
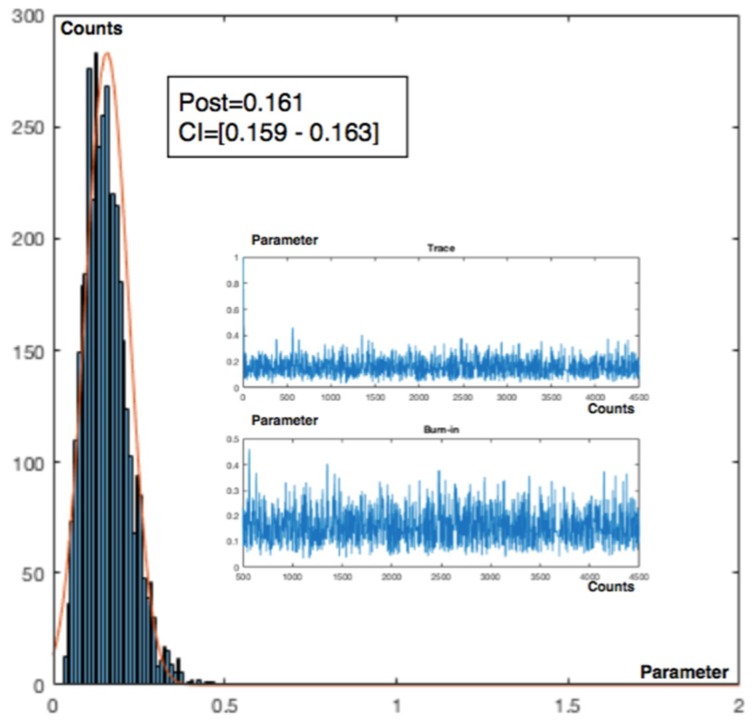
Metropolis–Hastings. Interval 4. Sampling n = 4500, burn = 500; 10 cycles. AR = 56%.

With the same 0.05 prior value and working with +/−1σ_post_ also in recurrent method with mean prior, the collected values are presented in Figure 21, and in Figure 22 the (MH) sampling with acceptance rate (AR) 56% at interval 4.

This model also presents out of limits at intervals 4, 7 and 8. The posterior is a normal distribution.

#### 4.2.5. Poisson–Normal Model

In the Poisson–normal model the posterior expression *f*(*p*/*data*)∝*g*(*data*/*p*)*·f*(*p*) with parameter *p* = λ is;

*f*(*λ*/*data*))∝*g*(*data*/*λ*)*·f*(*λ*)∝(*λy*)*^n^*/*n*! *exp* (−*λy*)∙1/*σ exp* (−(*x* − *λ*)^2^/2*σ*^2^ (7)

Being *y* the observed data and *x* the evolution of the parameter value.

**Figure 23:**
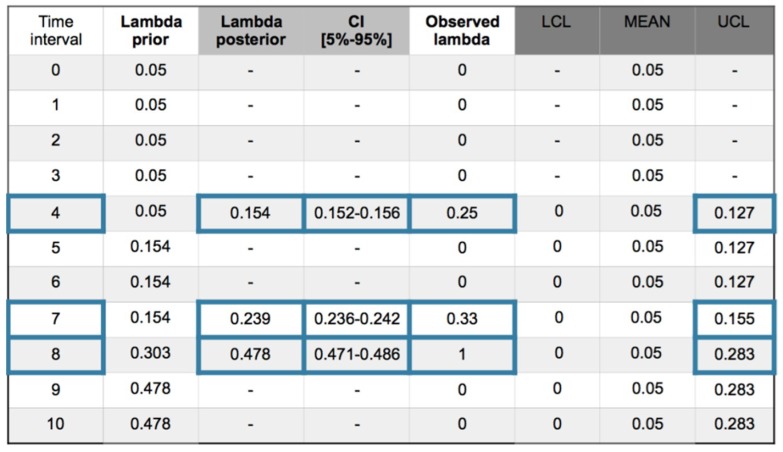
Poisson–normal model. Recurrent with mean prior method. Evolution of collected (ic’s) and safety barrier fails in 10 intervals. Confidence interval (CI) [5%–95%]. Out of limits framed blue.

**Figure 24:**
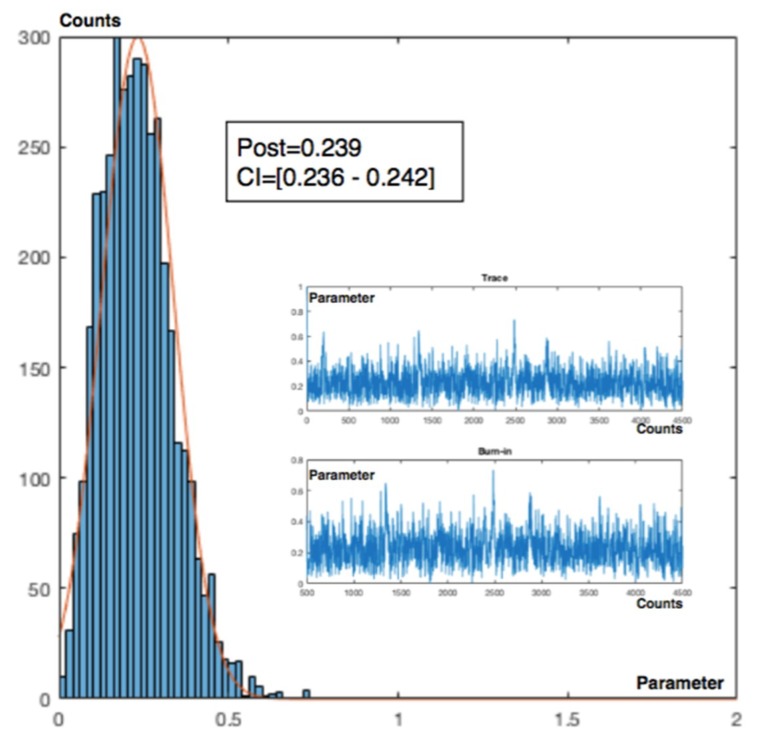
Interval 7. Sampling n = 4500, burn = 500; 10 cycles. AR = 57%.

With the same 0.05 prior value and working with +/−1σ_post_ in a recurrent method with mean prior, the collected values are presented in Figure 23, and in Figure 24 the (MH) sampling with acceptance rate (AR) 57% at interval 7.

This model also presents out of limits at intervals 4, 7 and 8. The posterior is a normal distribution.

#### 4.2.6. Analysis of the Mitigative Safety Barriers Observing End States

**Figure 25:**
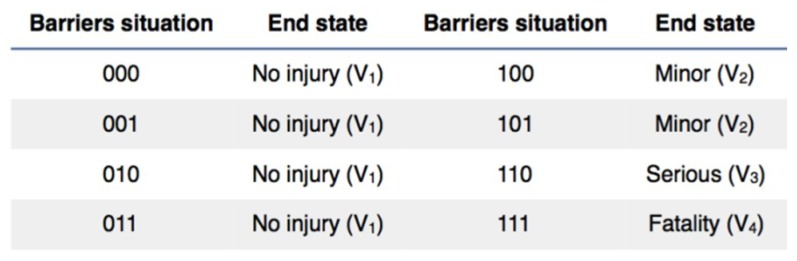
End states in function of the mitigative safety barriers situation.

If “0” is defined as a barrier is “correct” and active and “1” is in “fail” the possible end states are defined in Figure 25. Where “000” means that the first, second and third barriers are active and the end state is V_1_ = No injury; and the same logic applies to the rest.

**Figure 26:**
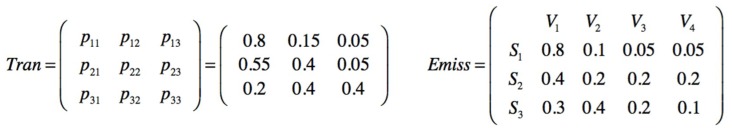
Prior transition and emission matrices for mitigative safety barriers and end states.

A prior transition matrix is defined for the three safety barriers. Being *p*_11_ the probability for the SF1 barrier to stay active in state 1; *p*_12_ the probability of transition from SF1 active to SF2 active because SF1 has failed; *p*_13_ is the probability of transition from SF1 to SF3 because SF1 has failed and also SF2 has failed, and so on. Additionally an emission matrix is defined to indicate the probabilities that a barrier S_n_ is active based on the observed end states (V_1_ V_2_ V_3_ V_4_), Figure 26.

**Figure 27:**
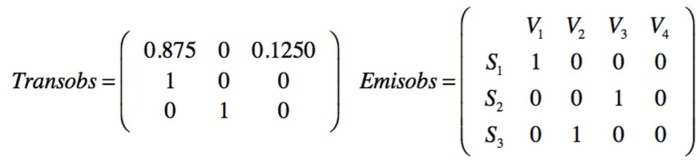
Posterior transition and emission matrices obtained from the observed sequence.

The observations are made by creating a group of ten following a first in first out(FIFO) order. Each new one is added to the group and the oldest one disappears. The observed sequence is seqobs = [1111112311] indicating that in the six first observations times no injury has been sampled, one minor injury in the seventh, one serious in the eighth, and no injury in the next two observations. Performing a Baum–Welch algorithm, the posterior transition and emission matrices are obtained from the observed data, Figure 27.

**Figure 28:**
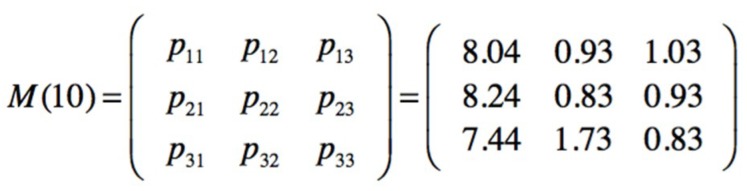
Number of visits for the posterior transition matrix.

Also, the relative occupations at steady state, after an infinite number of transitions, are obtained, being active SF1 = 80%, SF2 = 10% and SF3 = 10%. The number of visits or transitions to every state into the 10 observations are presented in Figure 28.

It is important also to know what will be the next passage, in observations intervals, from the SF1 and SF2 safety barriers to the most critical SF3 barrier. In this case is m_1_ = 8 and m_2_ = 9, meaning that, according to the observations, from SF1 a transition to SF3 can be produced in 8 intervals, and from SF2 to SF3 in 9 intervals.

**Figure 29:**
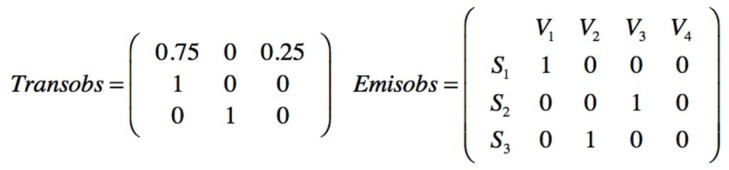
Posterior transition and emission matrices obtained from a new observation.

The posterior transition and emission matrices in Figure 27, can be the new prior in the next sampling, in this case a minor injury (V_2_) has been observed; then seqobs = [1111123112] and a new posterior for transition and emission matrices is obtained, Figure 29.

**Figure 30:**
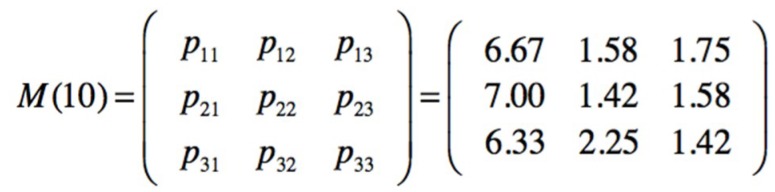
Number of visits for the posterior transition matrix.

With occupations at steady state SF1 = 67%, SF2 = 17% and SF3 = 16%, the number of visits or transitions are presented in Figure 30.

The next passage values for the third safety barrier are m_1_ = 4 and m_2_ = 5, meaning that from SF1 a transition to SF3 can be produced in 4 intervals, and from SF2 to SF3 in 5 intervals. The values are lower than the previous ones because the sampled observation has been for state V_2_, a minor injury, which reduces the number of no injuries, state V_1_ and makes the possible use of the third barrier more critical.

#### 4.2.7. Analysis of the End States Observing Mitigative Safety Barriers

**Figure 31:**
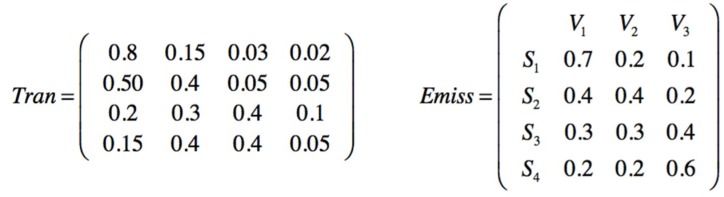
Prior transition and emission matrices for end states and mitigative safety barriers.

The prior matrices are defined as the transition matrix for the four end sates and the emission matrix indicating the probabilities to stay in an end state S_n_ being: S_1_ = No injury; S_2_ = Minor injury; S_3_ = Serious injury and S_4_ = Fatality; in function of the observed three active mitigative barriers (V_1_ V_2_ V_3_), Figure 31.

**Figure 32:**
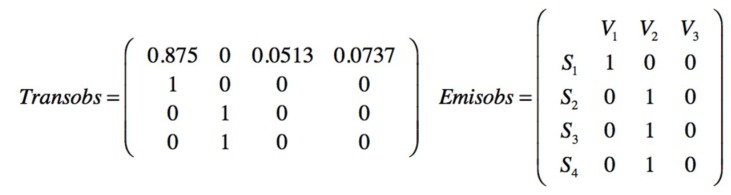
Posterior transition and emission matrices.

The sampling is obtained by sequentially observing the barriers, if the first is active, then its value is 1, if it fails and the second barrier is active, then the value is 2 and so on; a group of 10 observations is also maintained. The sampled sequence of the safety barriers is seqobs = [1111112211]. The posterior transition and emission matrices are presented on Figure 32.

**Figure 33:**
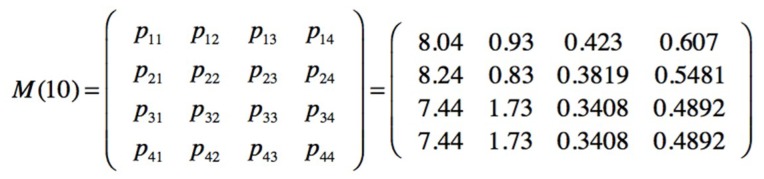
Number of visits for the posterior transition matrix.

With occupations V_1_ = 80%, V_2_ = 10%, V_3_ = 4% and V_4_ = 6%, and number of visits or transitions presented in Figure 33.

Showing a high number of visits to the end state 1 in concordance to with their occupations. The next passage values for the forth state, are m_1_ = 15, m_2_ = 16 and m_3_ = 17. Making a new observation and adding it to the group is seqobs = [1111122112]. The new posterior and emission matrices are obtained if the steady state occupations are S_1_ = 0%, S_2_ = 100%, S_3_ = 0% and S_4_ = 0%, indicating that in a steady state a high commitment is obtained in the use of the safety barrier SF2, due to the increase in the number of minor injuries corresponding to their associated final state S2.

**Figure 34:**
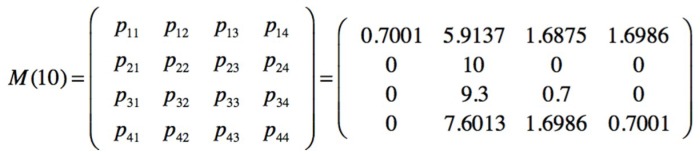
Number of visits for the posterior transition matrix.

The number of visits or transitions, are presented in Figure 34.

With next passage values for the fourth state; m_1_ = 2, m_2_ = 482 and m_3_ = 484. There is a correlation between the number of visits or transitions with the passage for the last barrier. In this case, in 10 intervals the transition *p*_44_ shows low stability in the fourth end state (Fatality) with a value *p*_44_ = 0.7, and the transitions *p*_24_ = *p*_34_ = 0 are indicating that there is no transition to the fourth state from the second and third and, therefore, it shows high passage intervals 482 and 484.

## 5. Discussion

The observations in which the risk parameter *p* is a frequency or ratio of risk causes requires the use of models based on Poisson, exponential and Weibull distributions that are those that have been used in this work. A recurring procedure has been used due to the characteristic of not having memory of this type of distributions, and each time interval begins as if it were new. The application of the gamma and normal distributions to characterize the risk parameter *p* is adequate when the variability and possible values are well collected, within the range of positive numbers, which this parameter can adopt.

The posterior distribution, because it is a reflection of the evolution of the prior, always takes the same distribution function.

The use of a control chart first requires, in every situation, previous tests to adjust the control limits. In this case the required accident rate value is very low. The control limits +/− 1sigma are restrictive in order to highlight the out of limits situation well in advance. Due to have a prior mean with a lower value near “0” and short upper limits has been possible to share the observations and the posterior mean with the same limit controls.

Despite the restrictive limits, the Poisson–gamma model showed just at the upper limit the first observation with value 0.25. As a characteristic the Bayesian inference softens the evolution of the statistical parameter based on the observations, for example, in the first observation at interval 4 with a value of 0.25, the Poisson–gamma model presents also a 0.25, showing an internal difference of a 4.5% into the group consisting of the exponential–gamma, exponential–normal and Poisson–normal but with a 60% difference with the Poisson–gamma. In the second observation at interval 7 with a value of 0.33, the inference models take values for a Poisson–gamma of 0.29, and the rest of models show an internal difference of 30% and a 135% difference with the Poisson–gamma. In the last observation with a value of 1, the Poisson–gamma shows a value of 0.38 and the exponential–normal with a value of 0.559 and Poisson-Normal with a value of 0.478 follow better this change to with the rest of inference models. At low changes the Poisson–gamma respond better and with important changes are better the Exponential-Normal and Poisson–normal.

The Metropolis–Hastings sampling has been done with a random simulation of 4500 values and removing (burning) the first 500 to guarantee the stability of the process, with a repetition cycle of 10 times, determining the average value and confidence intervals to obtain the representative posterior values. The resultant traces of the sampling processes also show a correct stochastic variability, with acceptance rates (AR) oscillating between 48% to 68% being necessary conditions to obtain a good sampling.

When initiation causes occur, at the same time information is being obtained on the status of the process and the different jobs, forcing a continuous review of its operation; for example, in the interval 4 due to a fault in a sensor that affects the hot pressing section, located in the sub-function of the SF1 mitigating safety barrier, it is a situation that requires verifying the same type of sensors. The same situation occurs when in the interval 7 there is a failure of self-control of work (JSC), then it is necessary to analyze the procedures and sub-functions that affect the design of the work, the environment of the operator, the characteristics of the operator, the interface of the system, procedures and information and design that affect workplaces. The last cause occurs in a failed rescue simulation that affects the SF3 safety barrier, which requires reviewing the performance of this procedure.

The hidden Markov chain procedure allows you to follow the situation of the mitigating barriers and obtain a map of your activity. The action of mitigating barriers is critical because when an accident occurs, they have to act with the highest probability that no failures will occur. However, it should be borne in mind that the method is an estimate of reality from the observations made allowing a quick view of which barrier is most used and likely to fail because it is showing high occupation. According to the experience gathered in the application of the Baum–Welch algorithm, the determination of the new transition and emission matrices from observations must have 10 values to obtain representative results.

If prior values for transition and emission matrices are not well known, the recurrent procedure adjusts the subsequent values according to the observations.

Collecting observations for the Bayesian inference or for the application of the hidden Markov chain can be carried out by the company’s own personnel, or by automated control systems, however human failures, in general, must be collected on site by personal observation.

A control chart process could be established without the need to associate it with a Bayesian inference; however, the objective of the new method, in addition to the graphic control of the evolution of risk, allowing simultaneous and immediate corrective actions, is having, on the one hand, the objective evaluation of a statistical parameter, which has been obtained from the observations that have been made through a formal procedure, and on the other hand, being able to have a continuous map of the functioning of the barriers of safety and the causes that affect the safety and health of workers.

The new method has strengths and weaknesses. The strong point is that the objective of the new methodology is that it is easy to implement and use to quickly gain an idea of the overall risk status of the process and of the worker’s situation; at the same time this is a weakness since it alone cannot be enough and must be complemented with other techniques. In addition, an important feature of the new method is that it is based on dynamic risk methodologies which, with respect to the quantitative risk analysis, allow the risk situation to be updated [87,126], and the weak points are based on the fact that by itself it is not a substitute for other methods. New methodologies are emerging as the use of the dynamic risk assessment and a decision-making trial and evaluation tool integrated into a Bayesian network for assessing the situations of leakage accidents [127], or a new assessment methodology based on the application of the FMECA including the economic valuation, [128] or the assessment of the domino effect using a graphical network of events (PETRI-NET) tool for discrete event treatment [129] or the general applications of the Bayesian networks and Petri-nets tools for risk assessment into the manufacturing and process industries [130] or the new techniques for the identification and monitoring of emerging risks over time [23,24]. These are new proposals that highlight the importance of risk assessment and the supporting role that the new SRC methodology can provide.

Future work is extensive, in a specific scenario the analysis can be carried out considering the entire industrial manufacturing process as a whole or analyzing more detailed areas. A sensitivity analysis can also be carried out determining which initiation causes (ic’s) or failures in safety barriers are more common and which areas within an industrial process have more incidence of risk.

## 6. Conclusions

From the review of existing methodologies, the dynamic methodologies are adapted to the need to have immediacy in the information. The new methodology (SRC) applies the characteristics of this group.

The bow-tie, which is an important element of the new methodology, was first generated by establishing a framework for the general treatment of risks, followed by adaptation to occupational accidents, which is applied without major changes in the treatment of the manufacturing plant case.

The content of the initiation causes (ic’s) and safety barriers have been defined and applied in the analysis of a occupational accidents in a plant for obtaining laminated paper and wood producing medium-density fiber (MDF) boards with melamine. The inference procedures applied in this case cover data observations belonging to Poisson, exponential and Weibull distributions and considering the risk statistical parameter under the evolution of gamma and normal distributions, showing that they are suitable for this type of analysis.

The application of the hidden Markov chain for the analysis of reactive safety barriers provides control for these types of barriers, since they are critical in their operation or failure in the event of an accident. The application of the hidden Markov chain for the analysis of reactive safety barriers provides control for these types of barriers, since they are critical in their operation or failure in the event of an accident. The tool, in its application to the case of the manufacturing plant, is suitable for obtaining information from the state of the different barriers or the situation of the different final states.

In conclusion, the SRC method is a formal method that allows us to meet the three characteristics of prevention, simultaneity and immediacy. It is applicable to the assessment of occupational hazards in different industrial and manufacturing scenarios, and offers an overview of the risk status in the simplest way possible and at the same time it is reliable, in accordance with the established control limits and providing warning in advance to be able to prevent risk.

This work can point towards future research actions considering its application in different types of industries and in project management, analyzing how it can complement other methodological tools.

## Figures and Tables

**Table 1:** Existing standards and regulations.

Std’s / Directives	Application	P	S	I
89/391/EEC	Occupational - basic. [10]	+	-	-
ISO 45001:2018	Implementation of a system of occupational health & safety (OH&S). [16]	+	-	-
NISHW	Spanish governmental organization of analysis and study for health and safety conditions in the workplace. [17]	+	-	-
98/24/EC, 2004/37/EC	Occupational - Chemicals and carcinogens concentration. [18,19]	+	-	-
ISO/IEC 31010:2019	Risk management process, based on a iterative cycle. Risk assessment based on identification, analysis and evaluation. General application of (QRA). [4,5,38]	+	-	-
PMBOK, PRINCE2	Documentation tailored forprojects. Design, Start, Direction, Planning, Execution, Control. [39,40]	+	-	-
CCPS	Layer of Protection Analysis (LOPA) methodology. A process deviation can lead to a hazardous consequence if not interrupted by an independent protection layer (IPL). Applied in chemical process. [14,41,42,43]	+	-	-
NORSOK 2010	Applied in the Norwegian petroleum industry, under the idea of Operational Risk Assessment, with the aim to follow the lifecycle of a project considering planning, execution and operation. [44,45,46]	+	-	-
2012/18/EUCOMAH 2015	European and British Control of Major Hazards for Seveso III Directive. Emergency plan withmajor accident prevention policy andinformation mechanism to authorities and population. A 5 years safety report. [47,48]	+	-	-
CPR18E	Netherlands advisory council of dangerous substances, and the old (Commissie voor de Preventie van Rampenthat, CPR). Applied in hazardous installations and transport analyzing the loss of containment events and the modeling of the associated flammable clouds, their dispersion and toxic effects. [49]	+	-	-
EN 16991:2018	European standards for chemical, power generation and manufacturing providing guidance for the inspection and risk evaluation in operations and maintenance. [50,51]	+	-	-

**Table 2:** Existing traditional models.

Models	Application	P	S	I
**Sequential**	Are representative of the Quantitative Risk Assessment (QRA) methodology regarding accidents as outcomes of a chain of discrete events or factors that take place in a temporal order. Analyzing causes and consequences of risk.	+	+/-	-
ETA	Event Tree Analysis. Consequence analysis. General application. [14]	+	+/-	-
FTA	Fault Tree Analysis. Causes of risks for human and technical systems. Applied in occupational risk analysis in the textile industry. [14,52]	+	+/-	-
BOW-TIE	Graphic including FTA and ETA models to represent causes, safety barriers, and consequence events. [14]	+	+/-	-
THERP	(Technique for Human Error Rate Prediction) a tool based on event-tree approach for evaluating human errors alone or in connection with equipment functioning, operational procedures and practices. [53]	+	+/-	-
FMEA	Failure Mode Effect Analysis. Step-by-step approach for identifying potential failures. [54]	+	+/-	-
Check list-What if	Systematic revision to find malfunctions and compliance with a list of requirements. [54]	+	+/-	-
FMECA	Failure modes, Effects and Criticality Analysis. Upgrade of the FMEA. The criticality is determined classifying the degree of potential failures. Case application for a toxic exposure to contaminants in a drug industry. [54,55]	+	+/-	-
RA	Reliability Assessment. Quantification of the probability of failure in a system. [56]	+	+/-	-
Block Diagrams	Graphical procedure describing the function of the system and showing the logical connections of components needed to fulfill a specified system function. [57]	+	+/-	-
HAZOP/HAZID	Technique for early identification of hazards usually applied in the design, the study is carried out by an experienced multi-discipline team using a checklist of potential hazards. [58]	+	+/-	-
EBM	Energy Barrier Model defining a safety barrier management and considering that an accident occur when hazards succeed to penetrate the safety barriers deficiencies. [59,60]	+	+/-	-
MORT	Management Oversight and Risk Tree. Root cause determination. Case for an elevator incident. [61]	+	+/-	-
SCAT	Systematic Cause Analysis. Causal analysis using a poster schematic which enables the identification of relevant corrective and preventive actions. [62]	+	+/-	-
STEP	Sequential Time Events Plotting. Identification of multiple causes in occupational accidents. [63]	+	+/-	-
MTO	Man Technology and Organization. Root causes in occupational work affected by the organization; practice; management; procedures and deficiencies in work environment. [64]	+	+/-	-
SOL	Safety through Organizational Learning. Event analysis in two steps: (1) description of the actual event situation, and (2) identification of contributing factors. Applicationin the nuclear industry. [65]	+	+/-	-
**Epidemiological**	Propagation of events is analogous to a disease spreading considering their distribution and determinants. Accidents are caused by latent events under epidemic context. Applicationin helicopter and road accidents. [66,67]	+	+/-	-

**Table 3:** Existing modern models.

Models	Application	P	S	I
**Systematic**	General risk framework based on the Rasmussen’s model using control theory concepts and considering that social climate is affected by government policy and budgeting, regulatory associations, organization, staff and the work operation systems for which their limitations and their interactions can allow preconditions for accidents. [68,69]	+	+/-	-
AcciMap	Cause event representation of the system interactions and how to control the hazardous processes originated into of the organizational and socio - technical system. [70]	+	+/-	-
STAMP	Systems Theoretic Accident Model.The systems are subject to external disturbances and can cause accidents due to physical, social and economic pressures and control failures in safety barriers. Human action supports part or all of the operation and actions of the system. A checklist is applied to identify control failures in safety barriers. [71,72,73]	+	+/-	-
CREAM	Cognitive Reliability and Error Analysis Method. Human performance is modeled to asses the consequences of the human errors. [74,75]	+	+/-	-
DREAM	Driving Reliability and Error Analysis Method. Application in driving accidents. [76]	+	+/-	-
FRAM	Functional Resonance Accident Model. As a result of the functional couplings appears resonance. The functional or basic processes in a risk scenario are identified, defining for each of them what are the inputs needed; the outputs produced; the needed resources (equipment, procedures, energy, materials and manpower); the controls to supervise, the preconditions to be fulfilled to carry the process and the time. The resonance can appear due to the variability in the dependence between processes. Application on aircraft, maritime and manufacturing. [77]	+	+/-	-
AEB	Accident Evolution and Barrier Function. Interaction between technical and human-organizational systems which may lead to an accident. The analysis needs work-team by engineers and human accidents specialists. [78]	+	+/-	-
**Cloud based**	Based on the FMECA . Perform a critical risk analysis based on the cloud by establishing a score based on expert knowledge. A case is presented for a gasification station. [79]	+	+/-	-
**Fuzzy based**	Application of fuzzy logical for define human behavior in risk situations.	+	+/-	-
HEART	Human Error and Assessment Technique.The reliability of any task can be modified by the influence of the Error Promotion Conditions (EPC). It is necessary to previously identify the tasks. For each task, with the help of a team of experts, a probability value of human error generation and the (EPC) that affect it and its relevance are defined. Fuzzy logic is applied to obtain a factor that modifies the probability of error. [53,80]	+	+/-	-
CREAM-BN	Upgrade of the systemic CREAM model.Human behavior has five components: strategic, tactical, opportunistic and scrambled. It is affected by common performance conditions (CPC) defined as: adequacy of the organization, working conditions, human-machine interface, operational support, availability of procedures, number of simultaneous objectives, available time, time of day, training and experience and quality collaboration. A Bayesian network and fuzzy logic are applied to determine the probability of human error. Scramble and opportunistic are the ones with the highest probability. Cases and examples from nuclear industry, aircraft transportation, manufacturing, retail and chemicals. [81]	+	+/-	-
**Formal based**	Accident causation is approached using probabilistic schemes and Bayesian networks to model the interaction between causes and effects.			
WBA	Why Because Analysis. Bayesian networks are applied considering that each component is a system is affected from the overall system environment. Application in transportation and aircraft accidents [82]	+	+/-	-
**Safety Barrier**				
PHPAM	Process Hazard Prevention Accident Models. Accidents are initiated by hydrocarbon release and propagation, and it is needed to establish safety barriers into five groups of prevention: release, ignition, escalation, harm and loss. Risk probabilities are evaluated before and after barriers implementation. [83]	+	+/-	-
SHIPP	System Hazard Identification Prediction and Prevention.Update of the initial probability of risk according to the actual data collected and application of Bayesian inference. [84,85]	+	+	-

**Table 4:** Existing dynamic models.

Models	Application	P	S	I
**Dynamic**	Uses sequential models and the Bow-tie graph approach, performing a Bayesian inference analysis to update the failure probabilities from the information collected of the accident precursors.	+	+	-
DyPASI	Dynamic Procedure for Atypical Scenarios Identification. Identification and assessment of the potential hazards based on information from atypical accident scenarios or situations, which are not captured by conventional HAZOP/HAZID techniques. [104,105]	+	+	-
Dynamic Risk Analysis	Analysis process as a step of the Dynamic Risk Assessment methodology being a quantitative modern approach in which the frequency of accidents are updated by the application of the Bayesian theory. [106]	+	+	-
Risk Barometer	For continuously monitor the risk of failure in safety barriers based on an existing Quantitative Risk Assessment (QRA) or a Dynamic Risk Assessment (DRA) and on the Barrier and Operational Risk Analysis (BORA). The safety barriers are analyzed through influencing factors, named Risk Influencing Factors, (RIFs), that are correlated to with theestimated probabilities of failure, followed by their visualization in an equivalent barometer graph. [107]	+	+	-
Dynamic Operational Risk Assessment	Markov and MonteCarlo chain simulations applied to analyze the incidence of events and causes in each component of a system process and its behavior. The method simulates the visits in each of the four states in which they can be found: normal operation; abnormal not detected; abnormal detected and under repair. [108]	+	+	-
